# PentaPlot: A software tool for the illustration of genome mosaicism

**DOI:** 10.1186/1471-2105-6-139

**Published:** 2005-06-06

**Authors:** Lutz Hamel, Olga Zhaxybayeva, J Peter Gogarten

**Affiliations:** 1 Department of Computer Science and Statistics, University of Rhode Island, Kingston, RI 02881, USA; 2 Department of Molecular and Cell Biology, University of Connecticut, Storrs, CT, 06269-3125, USA; 3 Department of Biochemistry and Molecular Biology, Dalhousie University, 5850 College Street, Halifax, NS B3H 1X5, Canada

## Abstract

**Background:**

Dekapentagonal maps depict the phylogenetic relationships of five genomes in a visually appealing diagram and can be viewed as an alternative to a single evolutionary consensus tree. In particular, the generated maps focus attention on those gene families that significantly deviate from the consensus or plurality phylogeny. PentaPlot is a software tool that computes such dekapentagonal maps given an appropriate probability support matrix.

**Results:**

The visualization with dekapentagonal maps critically depends on the optimal layout of unrooted tree topologies representing different evolutionary relationships among five organisms along the vertices of the dekapentagon. This is a difficult optimization problem given the large number of possible layouts. At its core our tool utilizes a genetic algorithm with demes and a local search strategy to search for the optimal layout. The hybrid genetic algorithm performs satisfactorily even in those cases where the chosen genomes are so divergent that little phylogenetic information has survived in the individual gene families.

**Conclusion:**

PentaPlot is being made publicly available as an open source project at .

## Background

Trees have a long history as models for the evolutionary history of organisms [1,2]. Lineage sorting and reticulate evolution have long been recognized as processes that make it difficult to infer species evolution from gene trees [3,4]. However, the extent of gene transfer between divergent species, particularly in case of microorganisms, has initiated a reassessment of the applicability of a tree-based concept for organismal evolution [5,6]. Individual genes coexisting in a present day genome can have very different evolutionary histories [7,8]. In particular, horizontal gene transfer is recognized as an alternative to *within lineage processes *like duplication and de-novo evolution of genes for an organism to acquire new properties [9]. Here we present a software tool, which computes dekapentagonal maps to depict the phylogenetic relationships of five genomes in a visually appealing diagram as an alternative to bifurcating trees. Dekapentagonal maps allow for the recognition of a plurality or majority signal and they can serve as a visual tool to pre-screen for putative instances of horizontally transferred genes (e.g., see [10]).

**Figure 1:**
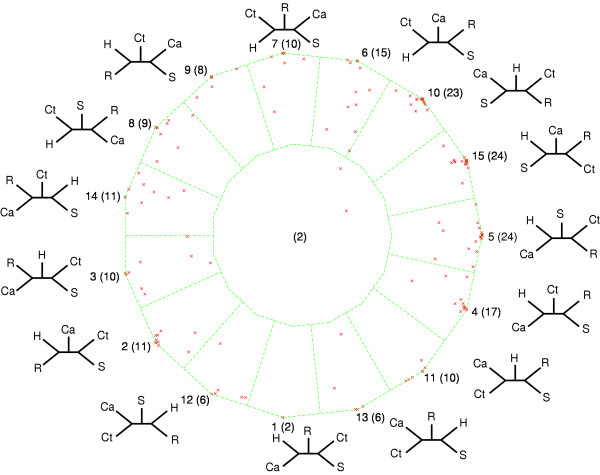
Dekapentagonal map for the analyses of five photosynthetic genomes: *Synechocystis *sp. (S), *Chloroflexus aurantiacus *(Ca), *Chlorobium tepidum *(Ct), *Rhodobacter capsulatus *(R) and *Heliobacillus mobilis *(H), based on posterior probabilities. Each point plotted within the dekapentagon represents a family of orthologous proteins – there are a total of 188 sets of orthologs common to the five genomes [26]). The dekapentagon is divided into zones of proximity to topologies: points that fall into one of the 15 zones that correspond to the 15 tree topologies favor either that topology most or several neighboring topologies, and points that fall into the single central zone represent unresolved relationships. The tree topology number (1 to 15) is given first, followed by the number of points per zone in parentheses. Abbreviations: Ca, *Chloroflexus aurantiacus*; Ct, *Chlorobium tepidum*; H, *Heliobacillus mobilis*; R, *Rhodobacter capsulatus*. This figure was previously published in [10].

Given five genomes we can characterize all possible phylogenetic relationships between the genomes with fifteen different unrooted tree topologies. One way to depict all fifteen relationships is to use a generalization of barycentric coordinates, so called dekapentagonal maps (see below and [10]). The support value vector for a gene family contains the posterior probabilities for each of the fifteen tree topologies given the aligned sequences, or the frequencies with which the fifteen different tree topologies are recovered from bootstrapped samples generated from the aligned sequences. The dekapentagonal map of five genomes depicts the support value vectors for all gene families that have a representative in each of the five genomes. The successful construction of dekapentagonal maps critically depends on an optimal layout of the fifteen different tree topologies along the fifteen vertices of the dekapentagon. Figure 1 is an example of a particular layout of the tree topologies along the dekapentagon's vertices (see [10] for detailed discussion of these analyses). The points within the diagram denote actual data support for particular tree topologies, with each point representing one family of orthologous genes [11,12]. The individual regions within the map demark areas of support for individual topologies. The region in the center of dekapentagonal map represents an area of no support for any particular topology. The resulting display facilitates recognition of frequently supported tree topologies (topologies #5, #10 and #15 in Figure 1) and their shared features (e.g., *Chlorobium tepidum *(Ct) grouping with *Rhodobacter capsulatus *(R)). The placement of a support value vector to the inside of the dekapentagon depends on how the fifteen topologies are laid out along the vertices. A gene family that has equal support for only two of the tree topologies will map to the periphery, if these two topologies occupy neighboring vertices, but it will map into the center, if the two topologies occupy opposing vertices. We define as optimal a layout of tree topologies along the vertices, if it minimizes the distance of the support value vectors from the periphery. This way an analysis of genomes related only through strict vertical inheritance will result in a cluster of points neighboring a single vertex; the horizontal transfer of several genes will result in points close to other not necessarily neighboring vertices (e.g. topology #2 in Figure 1), and tree topologies between which the data frequently cannot decide will be neighboring each other.

Computing the optimal layout of the tree topologies along the vertices of the dekapentagon presents a difficult optimization problem given the large number of possible layouts: (15-1)!/2 = 14!/2≈4*10^10 ^(only free circular permutations [13] are counted, and the arrangements that become equivalent by rotation or flipping of the dekapentagon are considered the same arrangements).

This setting seems ideally suited for optimization based on genetic algorithms [14]. At the core of the software tool presented here is the design and implementation of a hybrid genetic algorithm which computes optimal tree topology layouts along the dekapentagon vertices using demes in order to avoid premature convergence and which employs a local search strategy to complement the global search behavior of the crossover and mutation operators.

## Implementation

### Design overview

PentaPlot is written as a program, which is comprised of multiple processing steps implemented both in Perl and C++. The processing steps are hidden from the user by means of a master Perl script that ties all the processing steps together. In normal usage the user prepares a probability matrix (see software documentation for formatting details), which provides information about the support of particular tree topologies by the families of orthologous genes. This matrix is processed by the program into the visual dekapentagonal map as shown in Figure 1. The individual processing steps are as follows:

• Compute tree topology layout from probability matrix.

• Map the polar coordinates for orthologous gene family into a Cartesian coordinates.

• Summarize the number of genes, which fall into the individual zones that support particular tree topologies.

• Construct the dekapentagonal map.

The generated dekapentagonal map is available as an image, which either can be viewed in an interactive previewer or saved in the post-script and PDF formats. The implementation heavily relies on Wall's genetic algorithm C++ component library [15] and TeX [16].

PentaPlot also provides access to a number of tuning parameters for the construction of the maps, which are accessible via command line arguments:

• Iterations (default 50): Optimizing tree topology layouts with a genetic algorithm is a stochastic approach, therefore, in order to obtain some confidence in the computed solutions the solutions should be recomputed a number of times. This parameter controls how often the computation is to be repeated.

• Populations (default 10): A fundamental concept in genetic algorithms is the notion of a population. Here we apply a genetic algorithm that utilizes multiple populations at the same time in order to prevent premature convergence. This parameter specifies how many populations the algorithm should use.

• Population sizes (default 30): Population sizes are critical in genetic algorithms. If the population size is too small there will not be enough genetic diversity in the population to effectively explore the search space. If the population size is too large then a large amount of computation time might be wasted. This parameter controls the sizes of the populations.

• Maximum number of generations (default 500): The genetic algorithm as implemented in PentaPlot has an automatic stopping criteria built in based on 99% convergence within 50 generations, that is, if the performance of the best layout of the current generation T and the performance of the best layout of the generation T-50 are within 1% from each other then the genetic algorithm terminates. However, in particularly difficult optimization landscapes this convergence might never occur and the genetic algorithm might run forever (or at least for a very long time). To avoid this situation the maximum generation parameter allows the user to limit the number of generations the genetic algorithm is allowed to compute.

### Data preparation: The probability matrix

The first step in the phylogenetic analysis of genomes is the detection of sets of orthologous genes from the genomes making up the set of five genomes under consideration, i.e., genes that share common ancestry and are related through speciation and not gene duplication events. We use reciprocal top scoring hits as a criterion to select orthologous genes, i.e., each of the genes picks the other members of the quintet as the top scoring hit in a BLAST search [12,17,18]. While this selection scheme aims at detecting orthologous genes, the resulting sets can only be considerate to be putatively orthologous. While this selection scheme aims at detecting orthologous genes, the resulting sets only can be considered to be putatively orthologous. These gene sets include horizontally transferred genes (xenologs), especially those instances, where the transferred gene replaced its ortholog, synologs that were brought into a single genome through lineage fusion [19,20] and duplicated genes where one or the other paralog was lost in each the analyzed genomes [11]. When we refer to sets of orthologous genes in the rest of the manuscript we mean that those orthologs are putative. Once these putatively orthologous genes are detected, they are aligned and all possible unrooted tree topologies are evaluated (fifteen topologies for five genomes) and either their posterior probabilities or bootstrap support values are calculated (see [12] for details on methodology). Therefore, each family of putatively orthologous genes is associated with a 15-dimensional *support value vector*. This construction results in probability matrices where each row represents a family of orthologous genes and each column represents a particular unrooted tree topology. A value in a matrix represents the probability of support for a particular tree topology by a particular gene family. It is important to note that all the probabilities in one record have to sum up to one. Any other method that calculates support value vectors can be used to produce valid probability matrices. Please note that the construction of the probability matrix is a preprocessing step and is not included in the PentaPlot program.

### Mapping of probabilities into barycentric coordinate systems

Barycentric coordinate systems (coordinate systems based on centers of gravity) are most easily explained using triangles instead of generalized polygons. A simple probability matrix is shown in Table 1.

**Table 1:** A simple probability matrix (see Figure 1 and text for more details)

	*Tree #1 *(T_1_)	*Tree #2 *(T_2_)	*Tree #3 *(T_3_)
Support value vector for a set of orthologous genes	P_1_	P_2_	P_3_

**Figure 2:**
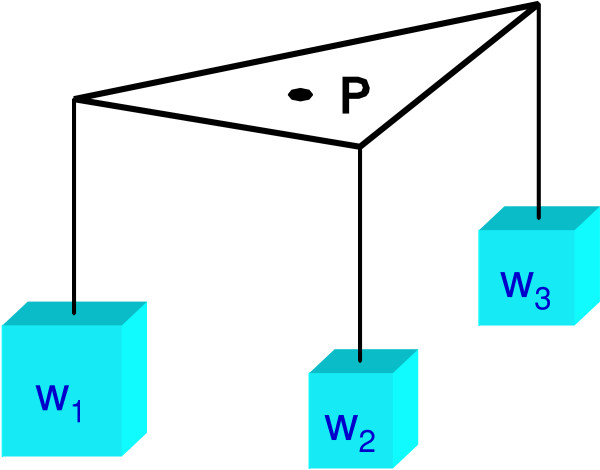
A barycentric coordinate system with three coordinates. See text and [12, 25] for more details.

**Figure 3:**
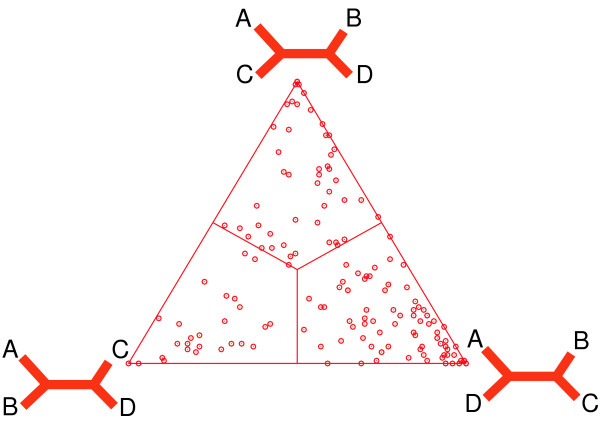
An example (modified from [11, 12]) of visualization of the support of three different unrooted tree topologies by multiple sets of orthologous genes from four genomes [12, 25].

With the tree topologies at the vertices of a triangle we can interpret the probabilities as weights at each corner of the triangle with the restriction that P_1 _+ P_2 _+ P_3 _= 1. We can then visualize this as shown in Figure 2. It is interesting to note that, given the particular assignment of the tree topologies to the vertices, the point P in the area of the triangle is completely defined by the support at each vertex, that is, the point P represents the center of gravity of this construction. We can now interpret the point P as a visual characterization of the support for each of the tree topologies by the set of orthologous genes in the example dataset above. If our dataset had more than one record (e.g., Table 2) we would see multiple points in the triangle, one for each set of orthologous genes. Figure 3 shows a visualization of a dataset with numerous orthologous genes and their support of three different tree topologies involving four genomes A, B, C, and D, respectively. The areas at the vertices demark the regions of support for the individual tree topologies.

**Table 2:** A three dimensional probability matrix that is used to calculate support value map depicted in Figure 3 where n is the number of orthologous genes considered. An analogous matrix with 15 columns can be constructed for 15-dimensional support value vectors.

	*Tree #1 *(T_1_)	*Tree #2 *(T_2_)	*Tree #3 *(T_3_)
Support value vector for a set #1 of orthologous genes	P_11_	P_12_	P_13_
Support value vector for a set #2 of orthologous genes	P_21_	P_22_	P_23_
...	...	...	...
Support value vector for a set #n of orthologous genes	P_n1_	P_n2_	P_n3_

**Figure 4:**
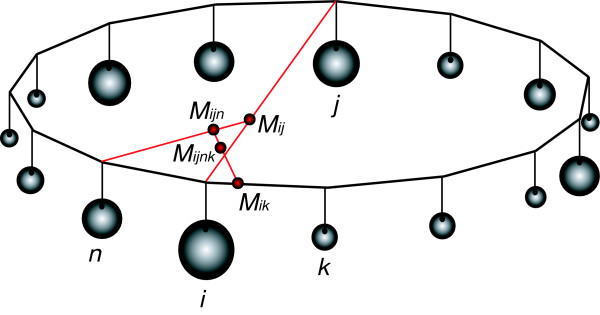
Schematic presentation of calculating and plotting support value vectors into a dekapentagon. Support values associated with each vertex are represented as weights attached to the vertices. Points *M *indicate locations of center of gravities of vertices that are mentioned in the index associated with each point *M*. See Implementation section for details of the calculation of the coordinates. This figure was previously published in [10].

We can generalize this to the case of the dekapentagon as shown in Figure 4. Similarly to the case of the triangle in Figure 2, we only show the construction of the center of gravity of a single set of orthologous genes. Again, the weights at the vertices represent the support of a particular tree topology by the set of orthologous genes. The points M_ij _denote the centers of gravity due to the weights indicated by the subscripts. When we include all the weights in this construction we will obtain a unique M that represents the center of gravity of all the weights for a particular arrangement of the weights along the vertices of the dekapentagon. Similar to the previous case we can interpret the center of gravity as a visual representation of the support for the tree topologies by this set of orthologous genes. Should there be multiple sets of orthologous genes in the dataset we will obtain multiple centers of gravity, one for each set of orthologous genes.

### Computing the center of gravity in a dekapentagon

Computing the center of gravity in a triangle is straightforward; however, the same computation in a dekapentagon deserves some remarks. To construct the center of gravity we place the dekapentagon into a Cartesian coordinate system with its origin coinciding with the center of the dekapentagon. Then the Cartesian coordinates (*x*_*i*_, *y*_*i*_) of a vertex *i *can be computed with the equations:



where *R *is the distance from origin to the vertex (equal for all the vertices due to the location of the origin of the coordinate system and here we assume that *R *is equal to 1), and 1 ≤ *i *≤ 15. For each pair of vertices *i *and *j *the coordinates (*x*_*M*_, *y*_*M*_) of the center of gravity *M*_*ij *_are calculated according to the law of the lever:



where *p*_*i *_and *p*_*j *_represent the support ("weights") at the vertices *i *and *j*, respectively. The process is repeated for all pairs of vertices, and then iteratively for all "intermediate" centers of gravities until only one pair of coordinates remains, which represents the center of gravity of the dekapentagon using all the "weights". The resulting coordinates of the dekapentagon's center of gravity do not depend on the order in which the masses are combined but they do depend on the particular arrangement of the support *p *along the vertices, i.e. on the way the 15 different tree topologies are assigned to the vertices. For example, one could envision a family of orthologous genes that supports only two of the fifteen possible tree topologies. If these two topologies were assigned to two opposing vertices, the support value vector would map to the center of the dekapentagonal map, indicating no particular support for any tree topology, whereas if the two topologies are assigned to neighboring vertices, the support value vector will map onto the periphery between these two vertices, revealing support for these two topologies over the other 13 alternatives. Therefore, it is crucial to compute a layout that moves the centers of gravity as close to the periphery as possible.

As mentioned above, in our case we not only compute a single center of gravity or barycentric point, but instead, given a data set with *N *orthologous genes we will compute *N *different barycentric points, one for each record in the data set.

### The layout algorithm

There are about 4*10^10 ^possible arrangements of topologies on a dekapentagon's vertices. An arrangement is considered optimal when the topologies are arranged at the polygon vertices in a way that maximizes the sum of the distances of all barycentric points from the center of the polygon. There are too many arrangements of topologies around the dekapentagon to search for the optimal arrangement exhaustively. Therefore, we used a heuristic search based on genetic algorithms [21].

In the genetic algorithm setup, each generation consists of a population of arrangements where each individual in a population encodes a particular mapping of the possible tree topologies identified by numerical identifiers (1 through 15) to vertices of the dekapentagon. The fittest individuals in a population maximize the sum of all distances of the barycentric points from the center of the polygon. As is typical in evolutionary computation, the genetic algorithm applies mutation and crossover operations to each successive generation of arrangements until an optimal solution is obtained [14]. Genetic algorithms today provide many different implementation strategies beyond the basic bit string genetic algorithm first developed by Holland [14]. We chose an array-based, hybrid genetic algorithm that uses demes to avoid premature convergence [15].

Genetic algorithms are good at finding approximate solutions in large search spaces but they are notoriously inefficient when it comes to fine tuning these solutions. By equipping a genetic algorithm with a local search strategy we avoid these problems. This is referred to as hybrid genetic algorithms [21] (sometimes also referred to as memetic algorithms [22]). Our hybrid genetic algorithm is summarized by the following pseudo code:

function evolve

create initial population

do

    // perform crossover and mutation

    population : = compute-new-population (population)

    best : = fittest-individual (population)

    optimized : = local-optimization (best)

    // if optimized is fitter than best replace best

    // with optimized in the population

    if (fitness (optimized) > fitness (best))

        replace (population,best,optimized)

until (stopping-condition)

return fittest-individual (population)

This algorithm is replicated over the demes giving rise to our hybrid deme genetic algorithm. It is noteworthy that we deviate from the standard notion of hybrid genetic algorithm slightly by only applying the local optimization function to the fittest individual of the population at each generation in each deme due to the computational cost of our local optimization: given the tree topology layout of the fittest individual, our local optimization strategy attempts to find an even better layout by systematically swapping tree topologies in the layout. The pseudo code of the local search heuristic follows:

function local-optimization (layout [1..15])

bestScore : = bestSource : = bestTarget : = -1

for s : = 1 to 14 do

    for t : = s+1 to 15 do

        swap-topologies (layout [s], layout [t])

        score : = scoreArrangement (layout)

        if (score > bestScore)

            bestScore : = score

            bestSource : = s

            bestTarget : = t

        endif

        undo-swap (layout [s], layout [t])

    endfor

endfor

swap-topologies (layout [bestSource], layout [bestTarget])

return layout

This procedure steps through all topologies in a layout and evaluates the score obtained by swapping each topology with all other positions. Here 'scoreArrangement' is a procedure that computes the barycentric coordinates for each record in the data set and the score is derived from the sum of all the distances of the barycentric points from the center of the polygon. Most likely we can improve the computational requirements of this local search procedure by sampling possible swaps in the layouts instead of trying them all [23].

Another key notion beyond the local search strategy is that we have to constrain the structure of the individuals in the populations in such a way that each individual can only encode legal sequences of topologies, that is, each individual can only encode layouts of topologies around the perimeter of the polygon that do not have repetitions. This is analogous to the term closure condition that arises in genetic programming where any term constructor combined with any other legal term constructor must give rise to a legal term [24]. Here we opted for an array representation where each position in the array denotes a vertex on the polygon. The contents at each array position denote a tree topology assigned to that vertex. Each individual is initialized in such a way that the tree topologies 1 through 15 are assigned to the vertices in such a way that there are no repetitions. In order to make this work the crossover and mutation operators have to preserve the uniqueness property of the topology layouts. Goldberg's PMX (partially matched crossover) operator [21] and Wall's swap mutation implemented in GALIB [15] fulfill our uniqueness requirement and have been implemented in PentaPlot.

## Results and discussion

We tested the design of our algorithm with four experiments of increasing difficulty [10]. Each experiment involved the comparison of five genomes. We applied both posterior probability mapping and bootstrap support value mapping [11,12,25] to two different genome quintets:

1. An inter-domain genome quintet consisting of representatives of all three domains of life: *Saccharomyces cerevisiae *(Y), *Rhodobacter capsulatus *(R), *Bacillus subtilis *(B), *Archaeoglobus fulgidus *(A), *Sulfolobus solfataricus *(S).

2. Bacterial genomes representing the five phyla that contain organisms with chlorophyll-based photosynthesis: *Chlorobium tepidum *(Ct), *Chloroflexus aurantiacus *(Ca), *Heliobacillus mobilis *(H), *Rhodobacter capsulatus *(R), *Sulfolobus solfataricus *(S).

The two datasets resulting from the first genome quintet each had 53 records, that is, 53 families of orthologous genes with one representative in each of the five genomes. The datasets resulting from the second genome quintet each had 188 records. Our investigation reported in [10] corroborated the layouts produced by our algorithm. The increase in difficulty in these experiments arises from the fact that (a) maximum likelihood mappings tend to produce barycentric points which lay close to the circumference of the polygon making it more difficult to discern an optimal layout and (b) the bacterial genomes contained a large number of orthologous genes, that is, there were a large number of barycentric points that needed to be considered during optimization.

We also compared the performance of our hybrid deme genetic algorithm to other genetic algorithm implementations such as the binary string genetic algorithm, the array based genetic algorithm, and a deme based genetic algorithm in each of these four experiments. The population size of the genetic algorithms in all the experiments for all genetic algorithms was 300 individuals. In the case of the deme configurations this population was distributed over 10 subpopulations. We used a convergence stopping criterion of 99% with a window of 50 generations. Typical runs lasted between 50 and 70 generations. The convergence percentages were computed by averaging the number of times a genetic algorithm computed exactly the same layout over the fifty runs. We postulate that a high degree of reproducibility indicates either a global optimal solution or a very strong local minimum, which can be considered a quasi-optimal solution. Given that the reproducible solutions found by the genetic algorithms were corroborated in bipartition analyses [10] we are confident that the genetic algorithms did converge on a global optimum. The results are summarized in Table 3.

**Table 3:** Application of the different genetic algorithms to four experiments with increasing difficulty (the percentages indicate reproducability of solutions over fifty runs).

***Experiments \ GA Type***	***Binary***	***Array***	***Deme***	***Hybrid Deme***
1) Inter-domain Bootstrap	0%	0%	100%	100%
2) Inter-domain ML Mapping	0%	0%	84%	84%
3) Bacterial Bootstrap	0%	0%	62%	72%
4) Bacterial ML Mapping	0%	0%	56%	72%

In the case of the binary string and array genetic algorithms it was interesting to see that we did not achieve reproducible solutions. Introducing demes into the genetic algorithms produced the most dramatic performance jump as can be seen from Table 3. In the case of the first experiment the performance jumped from 0% to 100% and dropped off with increasing difficulty of the experiments. The deme genetic algorithm performed well over the range of the experiments. However, in the fourth experiment it only converged on an optimal solution on every other run. The fourth column shows the performance of our hybrid deme genetic algorithm. We can see that it shares the performance characteristics of the deme genetic algorithm on the easier experiments but the performance of the hybrid deme genetic algorithm did not degenerate as fast with increasing difficulty of the experiments.

Tables 4 and 5 summarize the performance of the hybrid deme genetic algorithm using parameters other than the default parameters. In these experiments we applied our hybrid deme genetic algorithm to the maximum likelihood mapping of the bacterial genomes (our most difficult experiment). Table 4 shows the convergence behavior of the genetic algorithm given different number of populations and different sizes of the populations. Here, convergence is defined as above. What is most intriguing here is that bigger populations are not necessarily better. One possible explanation for this premature convergence might be the fact that the single best individual we are selecting in the local search for improvement does not have as much an impact on the large populations as it does in smaller populations. Thus, larger populations are more prone to early stagnation in their search. Table 5 highlights the fact that the deme idea is indeed very important to this algorithm in order to prevent premature convergence. As can be seen from both tables, our default values present a reasonable tradeoff between convergence behavior and computational complexity implied in larger number of populations or large populations.

**Table 4:** Investigating the genetic algorithm with different population and size settings.

**Pop. Number \ Pop. Size**	**10**	**20**	**30**	**50**	**80**	**100**	**300**	**500**
**1**	12%	24%	26%	34%	44%	44%	20%	10%
**2**	36%	28%	38%	42%	54%	52%	16%	12%
**3**	24%	34%	46%	70%	60%	56%	24%	12%

**Table 5:** Assuming population sizes of 30 and 50, investigating the behavior of the genetic algorithm with different numbers of populations.

**Pop. Number \ Pop. Size**	**30**	**50**
**2**	38%	42%
**4**	52%	68%
**8**	60%	78%
**10**	72%	88%
**15**	82%	88%
**20**	82%	88%

## Conclusion

Dekapentagonal maps provide an alternative to a single evolutionary tree to visualize phylogenetic relationships between organisms. Here we presented a tool, which computes such dekapentagonal maps given an appropriate probability matrix. The visualization critically depends on the optimal layout of unrooted tree topologies along the vertices of the dekapentagon. Given the large number of possible layouts, this represents a difficult optimization problem well suited for genetic algorithms. At its core our tool utilizes a genetic algorithm with demes and a local search strategy. The chosen optimality criterion moves the individual barycentric points representing orthologous genes as far to the periphery as possible. The resulting arrangement places tree topologies between which individual data sets frequently do not decide next to each other. The developed hybrid genetic algorithm performs satisfactorily even in those cases where the chosen genomes are so divergent that little phylogenetic information has survived in the individual gene families.

## Availability and requirements

• **Project name: **PentaPlot

• **Project home page: **

• **Operating system(s): **linux, kernel version 2.4.18 and above

• **Programming language: **Perl, C++, LaTeX

• **Other requirements: **Perl 5, BioPerl 1.4, LaTeX 2e, GAlib 2.4.5

• **License: **GNU GPL

• **Any restrictions to use by non-academics: **contact authors

## Authors' contributions

LH wrote the code for the PentaPlot program. OZ and JPG developed overall methodology for dekapentagonal mapping. OZ prepared probability matrices for analyzed genome quintets. All authors contributed equally to the manuscript preparation.
